# Feasibility of a Postpartum Web- and Phone-Based Lifestyle Program for Women with a History of Preeclampsia or Gestational Diabetes: A Pilot Intervention Study

**DOI:** 10.1089/whr.2023.0039

**Published:** 2023-07-18

**Authors:** Julie Horn, Marit Kolberg, Vegar Rangul, Elisabeth B. Magnussen, Bjørn Olav Åsvold, Hege B. Henriksen, Rune Blomhoff, Ellen W. Seely, Janet Rich-Edwards

**Affiliations:** ^1^ Department of Public Health and Nursing, Norwegian University of Science and Technology, Trondheim, Norway.; ^2^ Department of Obstetrics and Gynecology, Levanger Hospital, Nord-Trøndelag Hospital Trust, Levanger, Norway.; ^3^ Center for Oral Health Services and Research, Mid-Norway (TkMidt), Trondheim, Norway.; ^4^ Faculty of Nursing and Health Sciences, Nord University, Levanger, Norway.; ^5^ Department of Obstetrics and Gynecology, St. Olavs University Hospital, Trondheim, Norway.; ^6^ Department of Clinical and Molecular Medicine, Norwegian University of Science and Technology, Trondheim, Norway.; ^7^ Department of Endocrinology, Clinic of Medicine, St. Olavs Hospital, Trondheim University Hospital, Trondheim, Norway.; ^8^ Department of Nutrition, Institute of Basic Medical Sciences, University of Oslo, Oslo, Norway.; ^9^ Division of Cancer Medicine, Department of Clinic Service, Oslo University Hospital, Oslo, Norway.; ^10^ Department of Medicine, Division of Endocrinology, Diabetes and Hypertension, Brigham and Women's Hospital, Boston, Massachusetts, USA.; ^11^ Harvard Medical School, Boston, Massachusetts, USA.; ^12^ Department of Epidemiology, Harvard T.H. Chan School of Public Health, Boston, Massachusetts, USA.; ^13^ Department of Medicine, Division of Women's Health, Brigham and Women's Hospital, Boston, Massachusetts, USA.; ^14^ Connors Center for Women's Health and Gender Biology, Brigham and Women's Hospital, Boston, Massachusetts, USA.

**Keywords:** feasibility, gestational diabetes mellitus, lifestyle behavior, postpartum, preeclampsia, prevention

## Abstract

**Background:**

Women with a history of preeclampsia (PE) or gestational diabetes mellitus (GDM) are at increased risk of diabetes and cardiovascular disease (CVD) later in life. Increased awareness of pregnancy complications as early warning signs for CVD has called for postpartum primordial prevention strategies. The aim of this study was to evaluate the feasibility of a postpartum web- and phone-based lifestyle program promoting healthy lifestyle behaviors to women after a pregnancy complicated by PE or GDM.

**Materials and Methods:**

Women with a validated history of PE or GDM were invited to participate in a nonrandomized pilot intervention study 3–12 months after delivery. The intervention was delivered over 6 months. All participants received tailored lifestyle counseling by a registered dietitian and access to information material on healthy lifestyle behaviors on the study's website. After inclusion, participants were invited to three study visits at baseline, 3 months, and 6 months. Feasibility outcomes included assessment of recruitment, retention, and acceptability. Secondary outcomes were changes in lifestyle behaviors and cardiovascular risk factors.

**Results:**

Of the 207 women invited, 44 were enrolled in the feasibility study and 40 women completed the intervention, corresponding to a recruitment rate of 21% and a retention rate of 91%. At the 3-month study visit, 94.6% of participants reported they had used the website. A total of 41.7% of the participants reported that they had achieved their personal goals during the intervention period.

**Conclusions:**

This study suggested the feasibility and potential acceptability of a web- and phone-based lifestyle intervention for mothers with recent PE or GDM.

**Clinical Trial Registration:**

clinicaltrials.gov, www.clinicaltrials.gov, no. NCT03993145.

## Introduction

Cardiovascular disease (CVD) is the leading global cause of death in women, and female-specific risk factors have gained growing attention over the last few decades.^1^ Adverse pregnancy outcomes, including preeclampsia (PE) and gestational diabetes mellitus (GDM), have consistently been associated with increased risk for CVD later in life.^2,3^ Much of the increased risk of CVD in women with adverse pregnancy outcomes appears to be mediated by the development of established cardiometabolic risk factors such as chronic hypertension or type 2 diabetes arising in the months or years after pregnancy.^4–6^

Previous research concluded that most CVD-related deaths and CVD events may be avoidable through adherence to cardiovascular health behaviors, including nonsmoking, physical activity, healthy diet, and maintaining a healthy weight.^7–9^ Increased awareness of PE and GDM as early warning signs for CVD has called for postpartum primordial prevention strategies.^10,11^

According to focus group studies done among women with PE and GDM, many women were motivated to engage in healthy lifestyle changes in the postpartum period but expressed the need for tailored support to overcome multiple barriers, such as time constraints, fatigue, lack of professional support from health care providers, and the emotional impact of PE and GDM.^12–16^ So far, lifestyle intervention programs for women in the postpartum period are scarce.

The objective of this nonrandomized pilot study was to develop and investigate the feasibility of a lifestyle intervention to Norwegian women with recent PE or GDM, including assessment of recruitment, retention, and acceptability. Secondary aims were to estimate the preliminary impact of the intervention on lifestyle behavior and cardiovascular risk factors.

## Methods

**FIG. 1.**
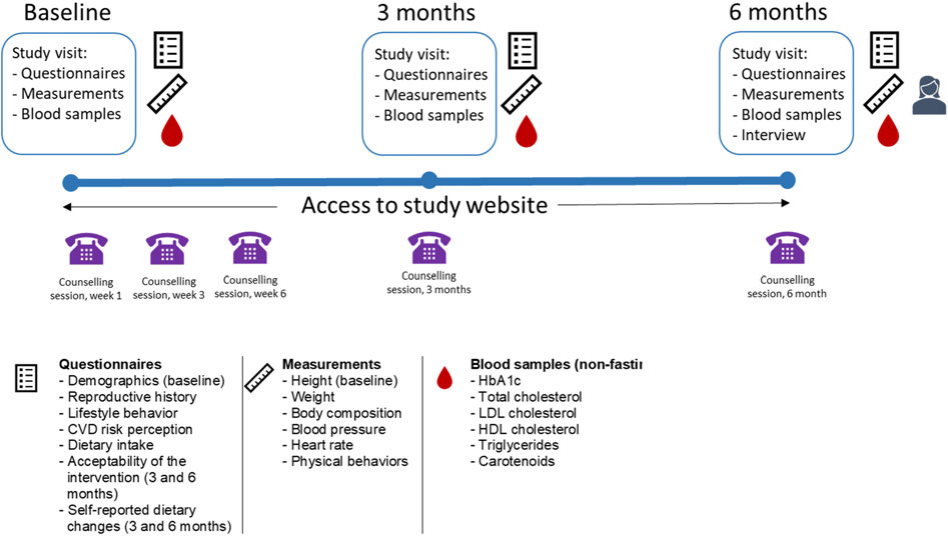
Schematic illustration of study design.

Mom's Healthy Heart (MHH) pilot study was a nonrandomized, single-arm intervention study (ClinicalTrial.gov identifier NCT03993145). All consenting women received an intervention addressing healthy lifestyle behaviors and were invited to three study visits at baseline, 3 months, and 6 months after inclusion. See Figure 1 for a graphic illustration of the study design.

### Recruitment/participants

We recruited participants between February and September 2020 from patients attending the Obstetric Departments at St. Olavs University Hospital in Trondheim, or Levanger Hospital (Nord-Trøndelag Hospital Trust) in Trøndelag County, Norway. No participants were included between March 12 and May 15 due to introduction of the first COVID-19 containment measures in Norway. Potentially eligible participants were women aged 18 years and older, who had given birth within the last 3–12 months and who had been registered with an International Classification of Diseases, Tenth Revision code, indicating PE and/or GDM in the electronic patient administrative systems of the two hospitals. Furthermore, due to transportation issues, women needed to reside within a 2-hour driving distance of one of the two hospitals. Diagnoses of PE and/or GDM were confirmed by medical record review by one of two gynecologists (J.H. and E.B.M.).

According to the current national guidelines, GDM was defined as onset of glucose intolerance in pregnancy with a 75 g 2-hour glucose tolerance test with fasting glucose ≥5.3–6.9 mmol/L or 2-hour value ≥9–11 mmol/L.^17^ PE was defined as *de novo* hypertension (≥140 mmHg systolic and/or ≥90 mmHg diastolic) after 20 weeks of gestation in combination with proteinuria (300 mg/24 hours or ≥1+ on the dipstick test) and/or signs of significant end-organ dysfunction.^18^

**FIG. 2.**
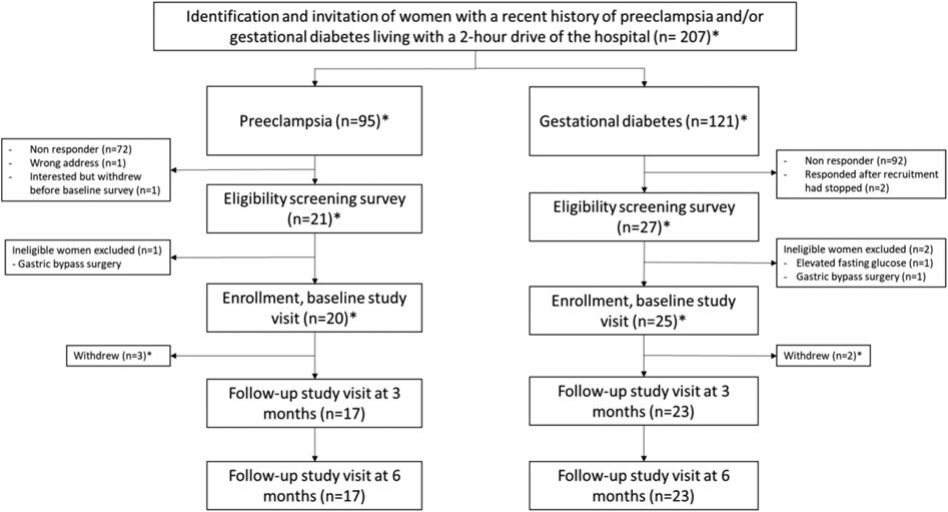
Study flow diagram according to pregnancy complication. *Nine of the invited women, two of the women who consented, one of the women who was ineligible, one of the women who was enrolled, and one of the women who withdrew had a history of both PE and gestational diabetes in their last pregnancy. These women are included in both groups in the flowchart. PE, preeclampsia.

Potentially eligible patients delivering between February 2019 and June 2020 received invitation letters by mail with an information sheet and consent form. No reminders were sent to nonresponders. Those who were interested in the study and returned the signed consent form were contacted by phone to assess exclusion criteria, including inability to speak and read Norwegian, diagnosis of chronic hypertension, diabetes mellitus type 1 or 2 or hypercholesterolemia, current use of blood pressure or cholesterol lowering medication, diagnosis of eating disorder, heart disease, stroke, or kidney disease, and previous gastric bypass surgery. Furthermore, women were ineligible for inclusion if they were currently pregnant, but we did not exclude participants who became pregnant during the study period. Figure 2 depicts the study flow diagram.

Potentially eligible women were invited consecutively until the desired sample size was achieved. The target sample size was 40 women, based on recruiting a large enough sample to assess the feasibility of the study and available resources of the study site.^19^

### Intervention

The intervention was delivered over 6 months and focused on improving adherence with the Norwegian food-based dietary guidelines (NFBDG) and increasing physical activity. We have incorporated various strategies for behavior change that are derived from a range of theoretical approaches.^20^ All participants received individualized phone-based counseling led by a registered dietitian (RD). The counseling sessions were scheduled for weeks 1, 3, 6, 12, and 24 (the 6-month study completion). Only two RDs were involved in the counseling, and the same RD followed the same women throughout the intervention period if possible. Principles from motivational interviewing were used as part of the counseling to explore and resolve the participants' ambivalence, and to identify motivation and barriers for behavior changes.^21^ During each counseling, personal, realistic, and achievable goals for diet and physical activity were set in agreement with the participant.

These goals were revised at each counseling, and potential challenges for behavior change were discussed. In addition to the counseling, tools and information on healthy diet, physical activity, and motivation to lifestyle changes were provided through access to the study website. The website included written information about diet and physical activity, audio-visual presentations, videos, examples for physical activity plans, healthy kid-friendly recipes, examples of portion sizes, and material for self-monitoring (Supplementary Figs. S1–S4). Recommendations for physical activity were adapted to the phase of early parenthood by flexible exercise plans including exercising with small children. Furthermore, we aimed to increase reflective motivation by enhancing participants' knowledge on healthy lifestyle behavior and understanding of the association between pregnancy complications and cardiovascular risk.

Nutritional advice and recommendations in MHH aimed to achieve adequate adherence with the NFBDG.^22^ These guidelines were developed to prevent chronic diseases, including CVD, in the general population. The guidelines emphasize a plant-based diet with high intake of fruits, berries, vegetables, whole grains, and fish and limiting the intake of red and processed meat, alcohol, salt, and sugar. The goal for physical activity was to achieve an average of at least 30 minutes of moderate physical activity per day or 150 minutes of moderate activity per week based on strength training and cardio/aerobic training.^23^

For overweight or obese women who aimed to lose weight, a stepwise weight loss up to 5%–10% during the intervention period was encouraged with a calorie deficit through less-energy dense food and regular physical activity. Women who were breastfeeding were advised a healthy diet and regular physical activity in line with the NFBDG. A slow weight reduction was recommended to ensure milk production with breastfeeding contributing to the calorie deficit.

Although family members were not actively included in the intervention, women were encouraged to share and discuss the information provided on the study website with their partners, other family members, or friends as social support.

### Data collection

Outcome measures were assessed at baseline, 3 months, and 6 months of follow-up study visits and included structured questionnaires and anthropometric, cardiovascular, and biochemistry measures. Study visits were scheduled between 8 am and 4 pm on weekdays. Measurements were performed by trained staff at the Clinical Research Ward, St. Olavs University Hospital in Trondheim or at the HUNT Research Centre in Levanger depending on participants' place of residence.

Women provided information on demographic data, reproductive history, smoking habits, sleep quality, CVD risk perception, and dietary intake. Furthermore, we collected information on the participants' motivation for initiating and maintaining lifestyle changes, the acceptability of the intervention, self-reported dietary changes (own and family members), achievement of personal goals, and experiences with perceived postpartum care through questionnaires (open and close-ended questions) at follow-up visits, and semistructured telephone interviews after completion of the intervention (Supplementary Table S2).

#### Anthropometric measures

Height and weight were measured with the participants wearing light clothes without shoes. Height was assessed at baseline only. Weight and body composition (Skeletal Muscle Mass [kg] and Muscle-Fat analysis [% and cm^2^]) was estimated at baseline, 3 months, and 6 months of study visits using bioelectrical impedance analysis (InBody 720/InBody 770, Cerritos, CA). Body mass index (BMI) was calculated as body weight in kilograms divided by the squared value of height in meters.

#### Biochemistry measures

Nonfasting blood samples were drawn at baseline, 3 months, and 6 months of study visits and analyzed for glycated hemoglobin (HbA1c), total cholesterol, low-density lipoprotein cholesterol, and high-density lipoprotein (HDL) cholesterol, and triglycerides at the laboratory of Levanger Hospital or St Olavs University Hospital. Carotenoids (lutein, zeaxanthin, β-cryptoxanthin, α-carotene, β-carotene, and lycopene) were analyzed in plasma by high-performance liquid chromatography with ultraviolet at the laboratory of Oslo University using an analysis method previously described by Bastani et al.^24^ Carotenoids serve as a biomarker for dietary intake of fruit and vegetables.^25^

#### Cardiovascular measures

Blood pressure and heart rate were measured in a sitting position using an automatic oscillometric method with cuff size adjusted to right arm circumference. Blood pressure and heart rate were measured three times with 1-minute intervals after 5–10 minutes of resting period. We used the average of the second and third measurement.

#### Diet quality

Dietary intake and adherence to the NFBDG recommendations in the intervention were measured using the semiquantitative short 63-item NORDIET-Food Frequency Questionnaire (NORDIET-FFQ) at baseline and at 3 and 6 months of follow-up study visits. NORDIET-FFQ assesses dietary intake over the previous 1–2 months. NORDIET-FFQ has been designed and validated at the Department of Nutrition University of Oslo and is used in an ongoing randomized clinical trial including patients diagnosed with colorectal cancer.^26,27^

Participants also self-reported on the general study questionnaire whether they or their family members have changed their dietary habits throughout the intervention period and were asked to describe changes in an open-text format.

#### Self-reported physical activity level

NORDIET-FFQ includes two questions regarding physical activity intensities, i.e., moderate and vigorous, given in minutes per day. It has been designed and validated to assess physical activity for the last 1–2 months.^28^

#### Measurement of physical behaviors

The study used a thigh-mounted (right thigh) triaxial accelerometer (Axivity, AX3) to measure physical behaviors (physical activity, sedentary behaviors, and sleep). The Axivity AX3 is a 3-axis logging accelerometer that can measure continuous time-series movement data (at a wide range of sample rates and durations). When attached directly to the thigh, the raw accelerometer was analyzed and we could classify physical behavior linked to both physical activity estimation (intensity, duration, and frequency) and posture detection (sitting, standing, and lying down). Thigh-worn accelerometers are validated for free-living behavior and therefore suitable for distinguishing between postures and shown high accuracy for capturing time spent standing, sitting, lying down, and therefore for estimating physical activity and sedentary time.^29,30^ The participants were instructed to wear the accelerometer for seven consecutive days (including water activities and sleep), both at baseline and follow-up study visits.

#### Analysis

Descriptive statistics are presented as mean and standard deviation (SD) or as numbers and percentages. Baseline characteristics between dropouts and study completers were compared using independent Student's *t*-test for continuous variables. Feasibility measures included assessment of recruitment and retention rates. Acceptability measures, including attendance of the counseling sessions, self-reported use of the study website, and personal goal achievement, were presented as numbers and percentages. Most lifestyle behaviors were not normally distributed and therefore presented as median and 5th and 95th percentiles. We conducted paired *t*-tests and Wilcoxon signed rank tests to compare lifestyle behaviors and cardiovascular risk factors before and after the intervention period. All statistical analyses were conducted using STATA 16 (StataCorp, College Station, TX).

To derive the physical behaviors from thigh-worn accelerometers, data were downloaded through Open Movement Software (version 1.0.0.42; Open Movement, Newcastle University, UK). The raw data were further processed and analyzed using a customized MATLAB program, ActiPASS, which determines the type and duration of different activities and body postures with a high sensitivity and specificity.^31^ The ActiPASS software is freely available upon request from the Occupational and Environmental Medicine, Department of Medical Sciences, Uppsala University. The detailed data analysis procedures in the ActiPASS software are described elsewhere.^30,32^ We included wear time between the first and last date and time recorded with a minimum of 20 hours of valid data per day.

### Ethical approval

This project was approved by the Central Norway Regional Committee for Medical and Health Research Ethics (reference no.: 2018/1803). All participants provided written informed consent.

## Results

Of the 207 women invited by mail, 49 (23.7%) provided written informed consent, of whom one woman withdrew before the baseline study visit and two women delivered the signed consent form after completion of the recruitment. We assessed 46 women for eligibility and included 44 women in the nonrandomized pilot study (21.3% of the invited women). Two of the women assessed for eligibility and one of the included women had a history of both PE and GDM in their last pregnancy (Fig. 2).

Participant characteristics at study entry are presented in Table 1. All women were married, or cohabitant, and most women were Nordic. Overall, the majority of participants had a high income and were highly educated with 84.1% of them having at least a college degree. Participants were on average 32.4 years (SD 5.4) old at baseline and 7.4 months (SD 3.0) postpartum. Cardiovascular risk factors at baseline are given in Table 2. None of the participants was currently smoking and the majority were overweight or obese. Compared with women with a history of GDM, although not statistically significant, women with a history of PE had slightly higher systolic and diastolic blood pressure (121 mmHg [SD 10] vs. 116 mmHg [SD 8] systolic and 79 mmHg [SD 11] vs. 77 mmHg [SD 7] diastolic), but lower HbA1c (33.6 mmol/mol vs. 36.3 mmol/mol).

**Table 1.** Baseline Characteristics of the Women at Study Entry

Characteristics	All participants (***n*** = 44)	Women with recent PE^a^ (***n*** = 20)	Women with recent GDM^a^ (***n*** = 25)
Age, years	32.4 (5.4)	30.6 (4.3)	33.6 (5.9)
Ethnicity			
Nordic	40 (90.9)	18 (90.0)	23 (92.0)
Other European	3 (6.8)	2 (10.0)	1 (4.0)
Asian	1 (2.3)		1 (4.0)
Education			
Secondary education	7 (15.9)	4 (20.0)	3 (12.0)
Lower tertiary education	16 (36.4)	9 (45.0)	8 (32.0)
Upper tertiary education	21 (47.7)	7 (35.0)	14 (56.0)
Partnership status			
Married or cohabitant	44 (100)	20 (100)	25 (100)
Household income			
<750,000 NOK	14 (31.8)	7 (35.0)	8 (32.0)
750,000–1,000,000 NOK	10 (22.7)	5 (25.0)	5 (20.0)
>1,000,000 NOK	19 (43.2)	8 (40.0)	11 (44.0)
Unknown	1 (2.3)		1 (4.0)
Employment			
Full-time	15 (34.1)	7 (35.0)	9 (36.0)
Part-time	7 (15.9)	5 (25.0)	2 (8.0)
Maternity leave	20 (45.5)	6 (30.0)	14 (56.0)
Unemployed	2 (4.6)	2 (10.0)	
Parity/Primiparous	23 (52.3)	16 (80.0)	8 (32.0)
Gestational age, completed weeks	37.1 (3.4)	35.6 (4.3)	38.4 (1.5)
Multiple pregnancy	2 (4.6)	1 (5.0)	1 (4.0)
Time since last birth, months	7.4 (3.0)	8.4 (3.3)	6.8 (2.8)

The data are presented as mean and SD or *n* (%).

^a^ One participant had a history of both PE and gestational diabetes in her last pregnancy and is counted in both columns.

GDM, gestational diabetes mellitus; PE, preeclampsia; SD, standard deviation.

**Table 2.** Cardiovascular Risk Factors at Study Entry

Characteristics	All participants (***n*** = 44)	Women with recent PE^a^ (***n*** = 20)	Women with recent GDM^a^ (***n*** = 25)
Smoking			
Current	0	0	0
Former, daily	6 (13.6)	2 (10.0)	4 (16.0)
Former, occasionally	5 (11.4)	2 (10.0)	3 (12.0)
Never	33 (75.0)	16 (80.0)	18 (72.0)
BMI, kg/m^2^	29.3 (5.5)	29.7 (6.4)	29.5 (5.4)
BMI categories			
Normal weight (18.5–<25 kg/m^2^)	9 (20.5)	5 (25.0)	4 (16.0)
Overweight (25–<30 kg/m^2^)	20 (45.5)	7 (35.0)	13 (52.0)
Obese (≥30 kg/m^2^)	15 (34.1)	8 (40.0)	8 (32.0)
WHR	0.81 (0.04)	0.82 (0.04)	0.81 (0.05)
Skeletal muscle mass, kg	28.4 (4.1)	28.3 (3.7)	28.6 (4.4)
Body fat percentage, %	36.9 (8.0)	36.8 (9.5)	37.4 (7.1)
Visceral fat area, cm^2^	133.4 (49.2)	136.3 (59.6)	135.8 (45.4)
Systolic BP, mmHg	118 (9)	121 (10)	117 (8)
Diastolic BP, mmHg	78 (9)	79 (11)	77 (7)
Heart rate, beats per minute	72 (13)	72 (14)	72 (12)
Total cholesterol, mmol/L	4.5 (0.7)	4.6 (0.8)	4.5 (0.6)
LDL cholesterol, mmol/L	3.0 (0.7)	3.1 (0.7)	3.0 (0.7)
HDL cholesterol, mmol/L	1.4 (0.4)	1.4 (0.5)	1.4 (0.3)
Triglycerides, mmol/L	1.4 (1.0)	1.4 (1.0)	1.4 (0.9)
HbA1c, mmol/mol	35.0 (3.3)	33.5 (3.0)	36.3 (3.0)
Total carotenoids, μmol/L	1.75 (0.5)	1.76 (0.5)	1.73 (0.5)

The data are presented as mean and SD or *n* (%).

^a^ One participant had a history of both PE and gestational diabetes in her last pregnancy and is presented in both columns.

^b^ Total carotenoids (lutein, zeaxanthin, β-cryptoxanthin, α-carotene, β-carotene, and lycopene) were analyzed among 43 women (one sample was excluded for not following the specified protocol for sample preparation).

BMI, body mass index; BP, blood pressure; HbA1c, glycated hemoglobin; HDL, high-density lipoprotein; LDL, low-density lipoprotein; WHR, waist-to-hip ratio.

### Adherence and acceptability

Of the 44 recruited participants, 40 completed the 6-month intervention, including all scheduled telephone-based counseling sessions. During the intervention period, four women decided to withdraw after 10–12 weeks, corresponding to a retention rate of 91%. Compared with study completers, women who dropped from the study were younger and had a higher BMI at baseline (Supplementary Table S1).

Self-reported usage of different parts of the study website, dietary changes, and personal goal achievement is presented in Table 3. At the 3-month study visit, 94.6% of study participants reported they used the website at least sometimes (less than once a week or more). Information on diet and physical activity was accessed at least once per week by 27.0% and 21.6% of the participants, respectively. However, a third (36.1%) never accessed the website information on motivation and self-efficacy, and only 5.6% accessed it once a week or more. At 6 months, self-reported usage of the website generally decreased, but still 86.5% of the participants reported they accessed the website information on diet at least sometimes (less than once a week or more).

**Table 3.** Self-Reported Website Usage, Dietary Changes, and Personal Goal Achievement

	3-month study visit	6-month study visit
How often have you accessed the study website for information on diet? (*n* = 37)		
Never	2 (5.4)	5 (13.5)
Sometimes	25 (67.6)	26 (70.3)
1 time per week	6 (16.2)	5 (13.5)
2–5 times per week	1 (2.7)	0
Almost daily	3 (8.1)	1 (2.7)
How often have you accessed the study website for information on physical activity? (*n* = 37)		
Never	9 (24.3)	10 (27.0)
Sometimes	20 (54.1)	21 (56.8)
1 time per week	4 (10.8)	5 (13.5)
2–5 times per week	2 (5.4)	1 (2.7)
Almost daily	2 (5.4)	0
How often have you accessed the study website for information on motivation and self-efficacy? (*n* = 36)		
Never	13 (36.1)	19 (51.4)
Sometimes	21 (58.3)	16 (43.2)
1 time per week	—	—
2–5 times per week	1 (2.8)	2 (5.4)
Almost daily	1 (2.8)	−
Have you changed your dietary habits throughout the study period? (*n* = 37)		
Yes	34 (91.9)	34 (91.9)
No	3 (8.1)	3 (8.1)
Have others in your family members changed their dietary habits throughout the study period? (*n* = 37)		
Yes	15 (40.5)	18 (48.7)
No	22 (59.5)	19 (51.4)
Have you achieved the personal goals that you have set yourself at the beginning of the study? (*n* = 36)		
Yes	—	15 (41.7)
No	—	19 (52.8)
Partly	—	2 (5.6)

The data are presented as *n* and percentages. Participants with missing values and those who became pregnant during study participation are excluded.

At the 6-month study visit, 41.7% of the participants reported that they had achieved their personal goals during the intervention period. Common self-reported reasons for not achieving personal goals were provided in an open-text format and included loss of motivation, not prioritizing themselves, health issues, and stress-related to work, family life, or the ongoing COVID-19 pandemic.

### Secondary outcomes

Results obtained by NORDIET-FFQ indicated a small decrease in meat consumption (87 g/d at baseline, 71 g/d at 3 months, 68 g/d at 6 months, *p* = 0.12) and an increase in whole grain products (83 g/d at baseline, 99 g/d at 3 months, 85 g/d at 6 months, *p* = 0.08), but did not suggest any significant changes in dietary habits throughout the study period (Table 4). However, more than 90% of women self-reported to have changed their dietary habits throughout the study period and nearly half of the participants reported that the dietary habits of their family members have changed as well (Table 3). In the open-text format, participants most commonly described that they reduced intake of sugar and fat and increased consumption of fish, whole grain products, fruits, and vegetables.

**Table 4.** Changes in Lifestyle Behaviors Throughout the Study Period (*n* = 37)

	Baseline Median (*P*_5_, *P*_95_)	3 months Median (*P*_5_, *P*_95_)	6 months Median (*P*_5_, *P*_95_)	***p*** ^*^
Estimated intake of food groups from NORDIET-FFQ				
Fruits, berries, and vegetables,^a^ g/d	328 (131, 946)	271 (124, 709)	264 (107, 814)	0.16
Fruit and berries,^a^ g/d	144 (50, 347)	126 (40, 324)	133 (29, 396)	0.29
Vegetables, g/d	155 (62, 607)	156 (73, 429)	156 (50, 513)	0.43
Unsalted nuts, g/d	7 (0, 32)	5 (0, 29)	4 (0, 27)	0.37
Whole grain products, g/d	83 (30, 229)	99 (33, 178)	85 (14, 166)	0.08
Fish, g/d	53 (0, 107)	51 (0, 176)	41 (0, 136)	0.58
Fatty fish, g/d	23 (0, 83)	23 (0, 131)	20 (0, 93)	0.21
Low-fat dairy products,^b^ g/d	22 (0, 124)	15 (0, 151)	24 (0, 92)	0.93
High-fat dairy products,^c^ g/d	14 (0, 146)	14 (0, 93)	15 (0, 75)	0.26
Unprocessed meat, g/d	54 (21, 150)	65 (0, 183)	44 (21, 171)	0.45
Red meat, g/d	87 (25, 181)	71 (25, 175)	68 (14, 181)	0.12
Processed meat, g/d	64 (4, 142)	58 (0, 175)	50 (13, 151)	0.14
Sugar-rich beverages, g/d	0 (0, 116)	0 (0, 116)	0 (0, 203)	0.29
Alcohol,^d^ g/d	1 (0, 20)	1 (0, 13)	0 (0, 12)	0.59
Physical activity from NORDIET-FFQ				
Moderate physical activity, minutes per day	16.3 (0.3, 52.1)	20.9 (1.4, 98.6)	12.8 (0.3, 72.6)	0.84
Vigorous physical activity, minutes per day	0 (0, 21.7)	3.6 (0, 21.7)	5.3 (0, 22.8)	0.08
Total physical activity, minutes per day	21.9 (0.3, 73.7)	24.2 (2, 114)	22 (0.9, 95.4)	0.66
Physical behaviors in terms of postures, step counts, and physical activity intensity (accelerometer measures)				
Time of sitting or lying, minutes per day	595 (415, 841)	605 (403, 817)	620 (408, 819)	0.48
Time of sitting, minutes per day	451 (247, 664)	465 (253, 633)	466 (270, 696)	0.27
Time of walking, minutes per day	79 (37, 133)	72 (32, 125)	65 (27, 136)	0.01
Time of walking fast,^e^ minutes per day	62 (26, 110)	56 (24, 105)	52 (18, 105)	0.01
Time spent in physical activity intensity				
Low intensity,^f^ minutes per day	307 (181, 461)	299 (177, 485)	300 (163, 490)	0.71
Moderate intensity,^g^ minutes per day	60 (25, 108)	53 (23, 99)	49 (18, 101)	0.01
Vigorous intensity,^h^ minutes per day	11 (2.4, 42.1)	8 (1.9, 37.1)	9 (2.0, 36.2)	0.06
Cadence,^i^ steps	10,237 (4,142, 17,710)	9,066 (3,917, 16,911)	8,617 (3,372, 16,539)	<0.01

Participants with missing values and those who became pregnant during study participation are excluded.

^a^ Includes juice, defined as maximum one portion of fruit = 100 g.

^b^ Includes low-fat dairy products (containing <20% fat), reduced-fat cheese (<17%), and lean milk (<1.5% fat).

^c^ Includes high-fat dairy products (containing >20% fat), high-fat cheese (>17%), and whole milk (>3.5% fat).

^d^ Alcohol intake was estimated using an alcohol factor, which was calculated based on the amount of alcohol (per 100 g of edible food) in a standard unit of beer, wine, or spirits: Beer with 4.7 vol-% alcohol: 3.8/100 = 0.038 (beer factor), wine with 10.2 vol-% alcohol: 10.2/100 = 0.102 (wine factor) spirits with 40 vol-% alcohol: 33.7/100 = 0.337 (spirit factor).

^e^ Time of walking with a cadence equal or higher than 100/min.

^f^ “Standing,” “Moving,” “Walking_Slow” (walking with a cadence lower than 100/min), and “Other” with no periodic movements and “Other” with periodic movements with a cadence lower than 100/min.

^g^ “Walking” (with a cadence between 100 and 135/min) and “Other” with periodic movements with a cadence between 100 and 135/min.

^h^ “Running,” “Cycling,” “Stair walking,” “Walking” with a cadence higher than 135/min, “Other” with periodic movement with a cadence higher than or equal to 135/min.

^i^ Total number of steps per day.

^*^ Wilcoxon signed-rank test, *p*-values for median intake of food groups and physical behaviors at baseline and 6 months.

NORDIET-FFQ, NORDIET Food Frequency Questionnaire.

Likewise, a small marginally significant increase in plasma carotenoids (0.14 μmol/L difference from baseline to 6 months, *p* = 0.05) indicated increasing fruit and vegetable intake (Table 5). Several women also described a higher awareness about healthy eating when answering the open-ended question on changes in dietary habits. Family member's dietary changes were in general described as similar to participants' dietary changes due to shared family meals. While results obtained by NORDIET-FFQ did not indicate any significant changes in moderate or vigorous physical activity, physical behaviors based on accelerometer data did suggest a slight decrease rather than any increase in physical activity throughout the study period (Table 4).

**Table 5.** Changes in Cardiovascular Risk Factors Throughout the Study (*n* = 37)

Characteristics	Baseline (***n*** = 37)	Changes at 3 months (3 months–baseline)	Changes at 6 months (6 months–baseline)	***p*** ^*^
BMI, kg/m^2^	28.8 (5.1)	−0.59 (1.01)	−0.62 (1.69)	0.03
WHR	0.81 (0.04)	0.002 (0.03)	0.004 (0.04)	0.45
Waist circumference, cm	88.1 (9.7)	−1.29 (3.91)	−2.84 (4.33)	<0.001
Hip circumference, cm	108.5 (10.8)	−1.93 (3.78)	−3.89 (4.97)	<0.0001
Skeletal muscle mass, kg	28.2 (3.8)	0.11 (1.42)	0.05 (0.92)	0.75
Body fat percentage, %	36.2 (7.9)	−1.44 (2.98)	−1.54 (2.81)	<0.01
Visceral fat area, cm^2^	128.5 (46.0)	−7.39 (13.02)	−7.79 (14.69)	<0.01
Systolic BP, mmHg	117 (9)	0.9 (7.6)	0.4 (8.3)	0.76
Diastolic BP, mmHg	78 (10)	−0.2 (6.2)	−2.0 (6.3)	0.06
Heart rate, beats per minute	71 (13)	−3.5 (10.9)	−2.4 (9.2)	0.12
Total cholesterol (mmol/L)	4.6 (0.7)	−0.16 (0.55)	−0.16 (0.53)	0.07
LDL cholesterol (mmol/L)	3.0 (0.6)	−0.13 (0.47)	−0.13 (0.46)	0.11
HDL cholesterol (mmol/L)	1.4 (0.4)	−0.10 (0.37)	−0.14 (0.37)	0.03
Triglycerides (mmol/L)	1.4 (1.0)	−0.22 (0.66)	−0.16 (0.68)	0.17
HbA1c (mmol/mol)	34.9 (3.3)	0.31 (1.78)	0.91 (2.25)	0.02
Total carotenoids,^a^ (μmol/L)	1.75 (0.5)	0.13 (0.39)	0.14 (0.39)	0.05

The data are presented as mean and SD. Participants with missing values and those who became pregnant during study participation are excluded.

^*^ Paired *t*-test for change from baseline to 6 months.

^a^ Total carotenoids (lutein, zeaxanthin, β-cryptoxanthin, α-carotene, β-carotene, and lycopene) were analyzed among 35 women. Two samples (one baseline sample and one 6-month sample) were excluded for not following the specified protocol for sample preparation.

Among 40 participants who completed the intervention, three women became pregnant between the 3- and 6-month study visits. A summary of the changes in cardiovascular risk factors in nonpregnant study completers is presented in Table 5. Most baseline values in anthropometric risk factors improved significantly, although modestly, over the intervention period. After 6 months, mean BMI decreased by −0.62 kg/m^2^ (*p* = 0.03), waist circumference by −2.84 cm (*p* < 0.001), hip circumference by −3.89 cm (*p* < 0.001), and visceral fat area by −7.79 cm^2^ (*p* < 0.01). In contrast, HDL cholesterol slightly decreased (−0.14 mmol/L, *p* = 0.03) and HbA1c slightly increased (0.91 mmol/mol, *p* = 0.02) from baseline to 6-month study visit. We did not observe any significant changes in the remaining cardiovascular risk factors.

## Discussion

Postpartum care services in Norway offer one free checkup 4–6 weeks after birth to assess the mother's physical and mental health and offer advice about contraception. Current guidelines for postpartum care of women with PE or GDM recommend healthy lifestyle counseling to reduce the future risk of T2DM and CVD, yet clinicians are limited in the time they have in clinical visits to provide adequate information and motivation. The findings of our pilot intervention study indicate that a web- and phone-based lifestyle intervention is a feasible supplement to postpartum care for women with prior PE or GDM.

Previous intervention studies for women with a history of PE or GDM reported varying recruitment rates, probably due to differences in recruitment strategies, health care systems, or risk perception. In the MHH intervention study, we observed recruitment rates ≈20% for both women with PE and GDM. Previous lifestyle intervention studies in the United States and Canada that identified potentially eligible women with a history of GDM from hospital databases reported higher recruitment rates of 40%–90%.^33–36^ In these studies, eligible participants were either actively invited during GDM clinic visits (during pregnancy or at 6 weeks postpartum) or actively contacted by a member of the study team up to 5 years after GDM diagnosis. In contrast, we sent a one-time mailing to the home.

Hutchesson et al. tested a web-based behavioral intervention for women with a history of PE in Australia and reported a recruitment rate of 11%, utilizing a similar recruitment strategy with letters sent to all women treated for PE within the last 4 years.^37^ Heart Health 4 Moms (HH4M), a US randomized intervention to reduce CVD risk in women with a history of PE, recruited participants nationally, largely through advertisements on websites and social media.^38^ The authors pointed out that participants who actively attempted to obtain information may have a healthier lifestyle and benefit less from the intervention than a more diverse clinic-based population. While we may have reached out to a more diverse group by actively inviting all women with a hospital diagnosis of GDM and PE, we may not have reached the optimal target population since our study participants mainly reported a high socioeconomic position.

This is in accordance with previously reported challenges related to the recruitment of study participants with low socioeconomic position into lifestyle interventions.^39^ It is possible that we might have had higher recruitment, particularly of women of lower socioeconomic position, if our recruitment strategy had used active recruitment from clinicians during face-to-face counseling and/or follow-up contacts by phone, mail, text, or other means.

Comparable with retention rates of previous studies,^34,35,37,38^ 91% of our study participants were retained until the 6-month study visit. Although few of the women reported accessing the website weekly or more, more than 90% did access it at least once, and 15% continued to access the site weekly or more, even at 6 months. All participating women completed all five scheduled counseling sessions with the dietician and most of them self-reported healthy dietary changes throughout the intervention period. This suggests that web- and phone-based interventions were feasible and acceptable for our study participants.

Future studies should, however, consider incorporating a more tailored approach for women with overweight or obesity to address the higher dropout rate of women with higher BMI. Measures to prevent the spread of COVID-19 have been introduced during the study period including temporary travel restrictions, social distancing, and restrictions on organized leisure time activities. These measures may have impacted the retention rate and participants' engagement in the intervention program.

Participants' self-reported changes in dietary habits on the general study questionnaire were in line with the observed small increase in carotenoids that suggested an increase in fruit and vegetable consumption. However, these findings were not in agreement with the dietary assessment based on NORDIET-FFQ. This may be due to social desirability bias, overreporting of dietary changes in open-text format, low statistical power, or smaller dietary changes that the NORDIET-FFQ was not able to capture.

While the slight decrease in objectively measured physical activity throughout the intervention period was somehow unexpected, it may be explained by a big between-subjects variability and study participants returning to work after maternity leave. Similar to previous studies on the assessment of physical activity, we observed that self-reported data differed from objective measures of physical activity.^40^ Whereas objective measures based on accelerometer data suggested a decrease in physical activity, self-reported data did not indicate any significant changes in physical activity throughout the study period. A lack of a clear trend among the differences between the self-report method for assessing physical activity and the more robust direct method is of concern, especially if trying to establish whether the measures could be used interchangeably.

There are several possible explanations, and the discrepancy may be due to participants overreporting physical activity. Another probable reason may be that accelerometers recorded all daily physical behavior including physical activity, sedentary behavior, and sleep, whereas self-reported data only included moderate and vigorous physical activity. Our findings demonstrate the inherent difficulty self-report measures possess when trying to accurately capture data at various levels of exertion. Compared with objective measures, self-report methods appear to estimate greater amounts of higher intensity (*i.e.,* vigorous) physical activities than intensities at low-to-moderate levels.^41^ At last, low-moderate level activities such as brisk walking may actually be underreported.^42^ This underlines the importance of including objective measures of physical activity in future studies.

We observed a moderate improvement of several anthropometric risk factors. However, due to the nonrandomized design, it is important to recognize that MHH only assessed changes within the group of women who received the intervention. We were not able to compare changes in cardiovascular risk factors among study participants to women who received usual care. Onyango et al. examined postpartum weight change among women in the World Health Organization (WHO) Multicentre Growth Reference Study (MGRS).^43^ The authors reported that Norwegian mothers on average lost weight during the first year postpartum, and postpartum weight loss was most apparent during the first 6 months postpartum.

Our study participants were on average recruited 7 months postpartum and thereby after the period of biggest weight loss. Compared with Norwegian mothers in the MGRS, our study participants had a higher BMI and several studies have shown that women with obesity have lower postpartum weight losses than women with normal weight.^43,44^ Supporting that our results of weight loss could, in part, be explained by the intervention, two randomized trials for women with recent GDM reported that women assigned to the intervention arm were more likely to reduce postpartum weight retention than women in usual care.^34,45^ In HH4M, however, no differences in weight reduction were found between the intervention and control arms.^38^

The small sample size and the nonrandomized design are limitations for interpreting the results. Nevertheless, the data provide valuable insight into changes in cardiovascular risk factors during the intervention period. Acceptability has been assessed quantitatively by using attendance of the counseling session, self-reported use of the study website, and personal goal assessment, but not by directly asking whether participants were satisfied with the intervention. Furthermore, qualitative data analyses may provide a better understanding of the participants' perceptions of acceptability.^46^ Several participants described that they have assessed the study website together with their partners and that family members have changed their diet due to shared family meals. Future intervention studies could consider the potential of a more active involvement of women's partners to increase participants' motivation for engaging in lifestyle behaviors.

Our study population was relatively homogenous, limiting the generalizability of our findings. In addition, women who lacked sufficient Norwegian language skills were not eligible for inclusion in the study. Furthermore, sending reminders to nonresponders or using other recruitment strategies might have increased the response rate especially among less motivated women. Future interventions should focus on broader overall recruitment, perhaps with enhanced outreach to women of lower socioeconomic position and education, who may benefit the most from the intervention.

## Conclusion

In summary, this pilot intervention study supports the feasibility and acceptability of a web- and phone-based lifestyle intervention for women with a history of PE or GDM and suggests that postpartum lifestyle interventions may contribute to improve new mothers' cardiometabolic health. Our experience indicates that future studies may benefit from more focus on effective recruitment strategies for women with a low socioeconomic position to decrease socioeconomic health inequalities. Larger, randomized controlled trials are needed to examine whether postpartum web- and phone-based lifestyle interventions improve health and whether eventual changes are sustained over time.

## Supplementary Material

Supplemental data

Supplemental data

Supplemental data

## Abbreviations Used

BMI: body mass index
BP: blood pressure
CVD: cardiovascular disease
GDM: gestational diabetes mellitus
HbA1c: glycated hemoglobin
HDL: high-density lipoprotein
HH4M: Heart Health 4 Moms
LDL: low-density lipoprotein
MGRS: Multicentre Growth Reference Study
MHH: Mom's Healthy Heart
NFBDG: Norwegian Food-Based Dietary Guidelines
NORDIET-FFQ: NORDIET Food Frequency Questionnaire
PE: preeclampsia
RD: registered dietitian
SD: standard deviation
WHO: World Health Organization
WHR: waist-to-hip ratio
