# Supplementary material for: Feasibility of a Postpartum Web- and Phone-Based Lifestyle Program for Women with a History of Preeclampsia or Gestational Diabetes: A Pilot Intervention Study
Source: Womens Health Rep (New Rochelle). 2023 Jul 18;4(1):345–57. doi: 10.1089/whr.2023.0039 (PMC10357112; doi:10.1089/whr.2023.0039)
Supplement: Supplemental data [file Suppl_FigS1-S4.docx]

**Supplementary Figure S1. Screenshot of the study website.**


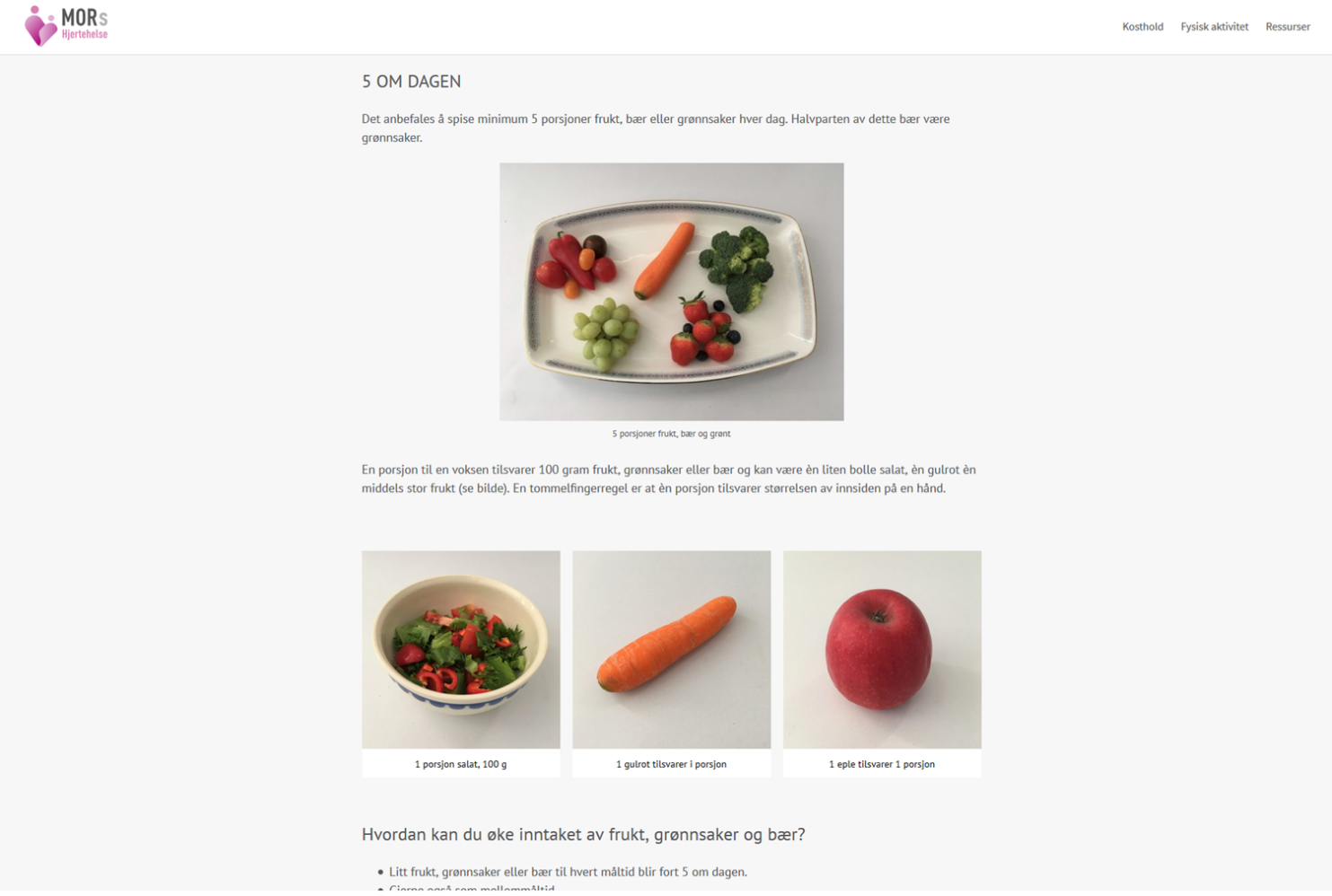


Photographs: Marit Kolberg

**Supplementary Figure S2. Screenshot of the study website.**


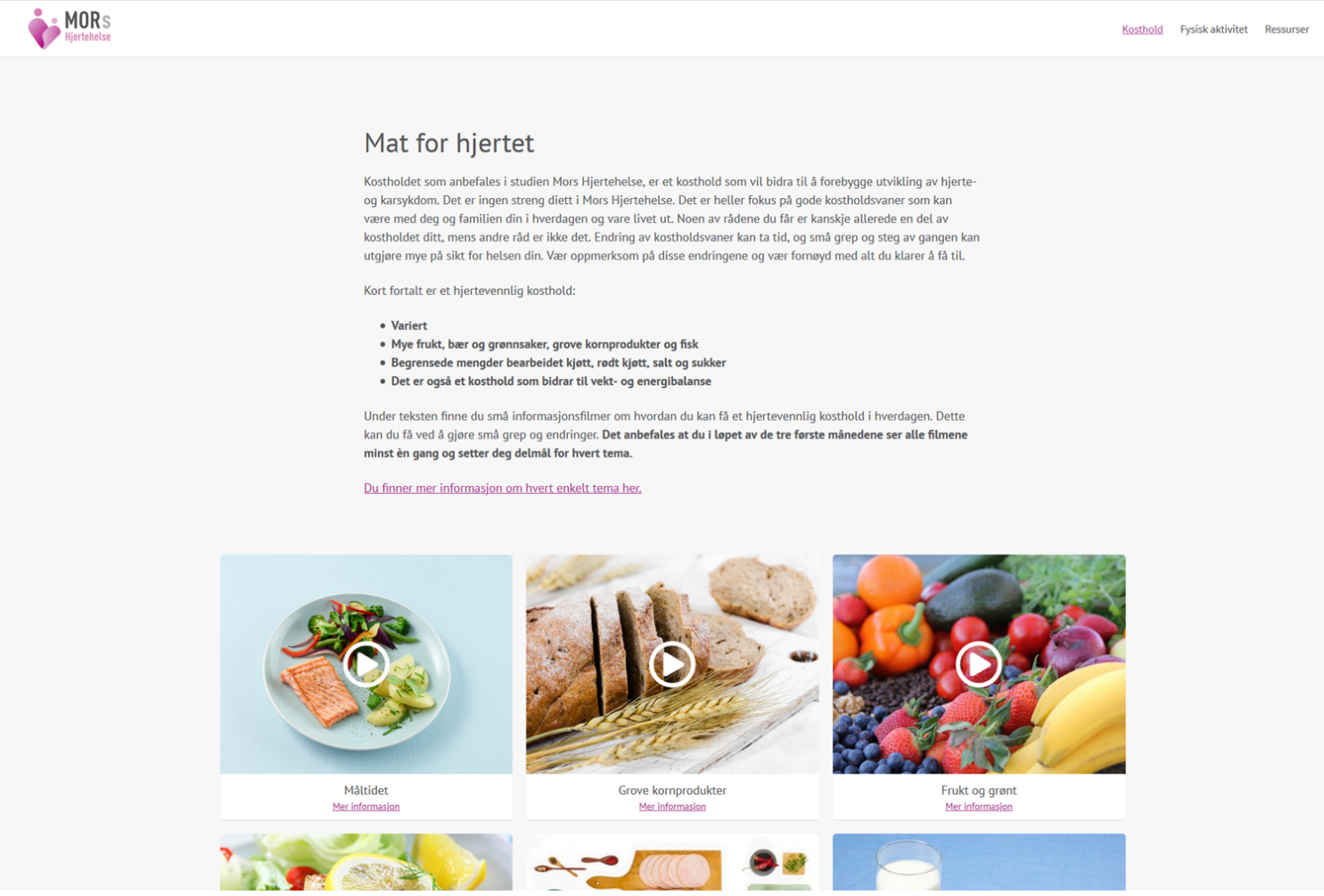


Illustrations and photographs: Aina Hole/Norwegian Directorate of Health,Colourbox, Marit Kolberg, Lisa Westgaard / Tinagent

**Supplementary Figure S3. Screenshot of the study website.**


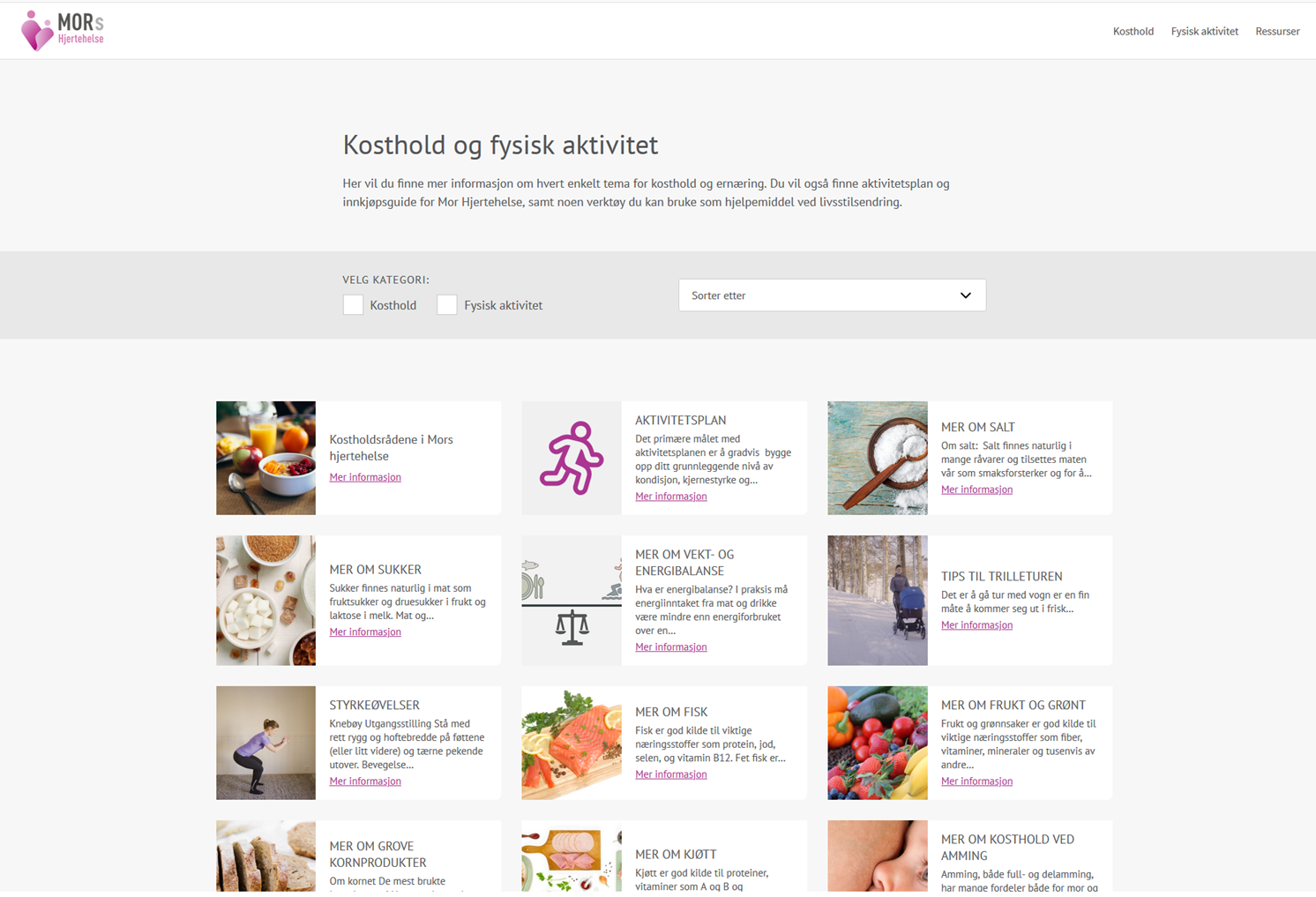


Illustrations and photographs: Colourbox, Lowri Rees, Lisa Westgaard / Tinagent, Aina Hole/Norwegian Directorate of Health, Marit Kolberg

**Supplementary Figure S4. Screenshot of the study website.**


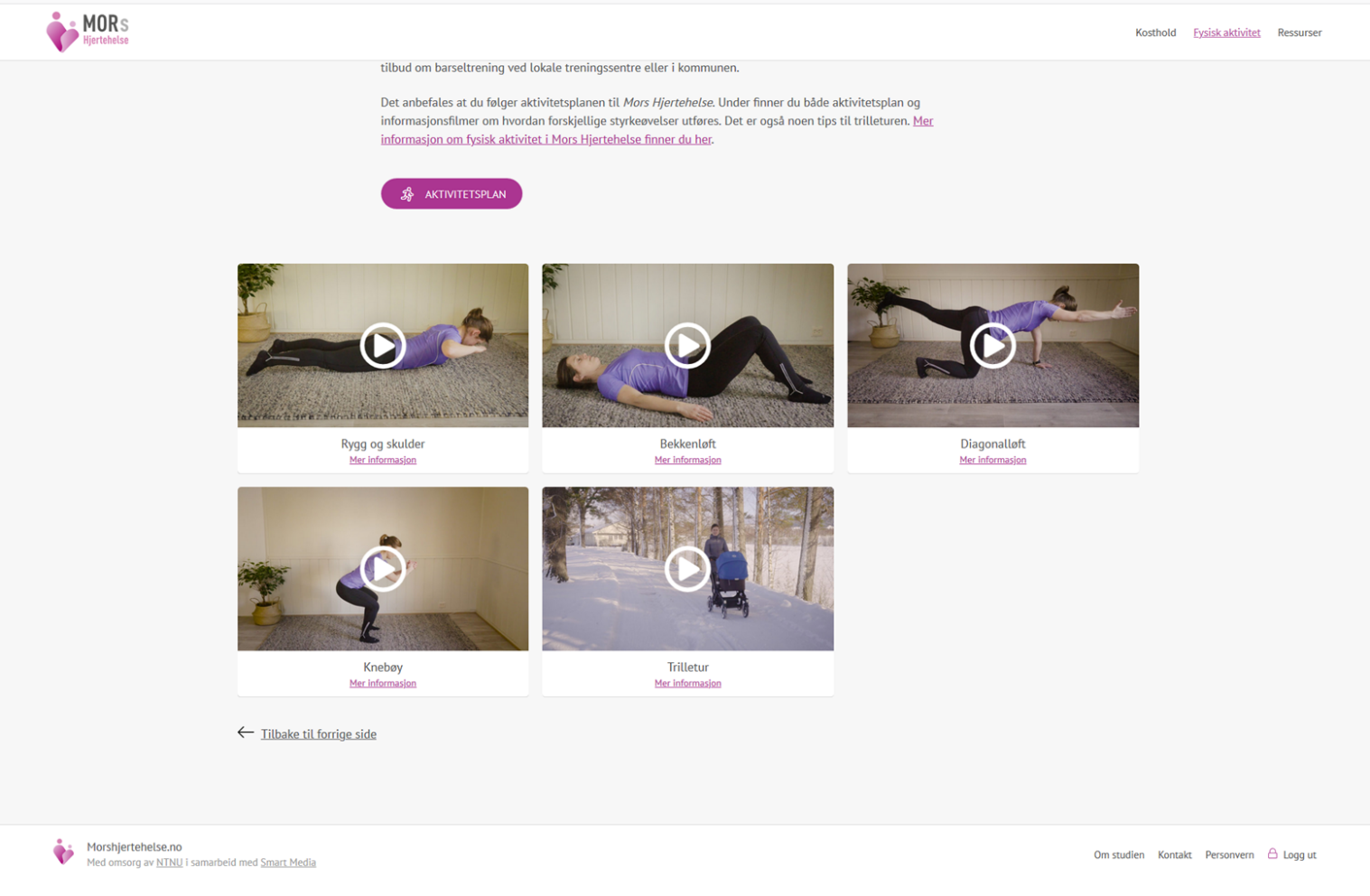


Photographs: Lowri Rees
