# Supplementary material for: Feasibility of a Postpartum Web- and Phone-Based Lifestyle Program for Women with a History of Preeclampsia or Gestational Diabetes: A Pilot Intervention Study
Source: Womens Health Rep (New Rochelle). 2023 Jul 18;4(1):345–57. doi: 10.1089/whr.2023.0039 (PMC10357112; doi:10.1089/whr.2023.0039)
Supplement: Supplemental data [file Suppl_TableS2.docx]

| **Supplementary Table S2. Schedule of assessments** | | | |
| --- | --- | --- | --- |
|  | Baseline study visit | 3 months study visit | 6 months study visit |
| Assessments |  |  |  |
| Demographics | X |  |  |
| Work | X | X | X |
| Obstetric history | X | X | X |
| Family history of CVD | X |  |  |
| Smoking | X | X | X |
| Dietary changes | X | X | X |
| Breastfeeding history | X | X | X |
| Sleep quality | X | X | X |
| Postpartum depression^1^ | X | X | X |
| Social support | X | X | X |
| Healthy lifestyle self-report | X | X | X |
| Diet quality, NORDIET-FFQ | X | X | X |
| Self-report physical activity, NORDIET-FFQ | X | X | X |
| Health care contact | X | X | X |
| Information CVD risk | X |  |  |
| CVD risk perception | X | X | X |
| Use of study website |  | X | X |
| BMI | X | X | X |
| Bioelectrical impedance analysis | X | X | X |
| Blood pressure | X | X | X |
| Heart rate | X | X | X |
| Waist and hip circumference | X | X | X |
| Total cholesterol^2^ | X | X | X |
| LDL cholesterol^2^ | X | X | X |
| HDL cholesterol^2^ | X | X | X |
| Triglycerides^2^ | X | X | X |
| HbA1c^2^ | X | X | X |
| Carotenoids^2^ | X | X | X |
| Physical activity sensor, AX3 Axivity | X | X | X |
| Interview^3^ |  |  | X |
| ^1^ Edinburgh postpartum depression scale; ^2^ non-fasting blood samples; ^3^phone interview after completion of the 6 months study visit  BMI=body mass index; BP=blood pressure; CVD=cardiovascular disease; HbA1c=glycated hemoglobin; HDL=high-density lipoprotein; LDL=low-density lipoprotein | | | |
