# Supplementary material for: Feasibility of a Postpartum Web- and Phone-Based Lifestyle Program for Women with a History of Preeclampsia or Gestational Diabetes: A Pilot Intervention Study
Source: Womens Health Rep (New Rochelle). 2023 Jul 18;4(1):345–57. doi: 10.1089/whr.2023.0039 (PMC10357112; doi:10.1089/whr.2023.0039)
Supplement: Supplemental data [file Suppl_TableS1.docx]

| **Supplementary Table S1. Baseline characteristics and cardiovascular risk factors at study entry for dropouts and completers** | | | | |
| --- | --- | --- | --- | --- |
| Characteristics |  | Dropouts  (n=4) | Women who completed the study (n=40) | p-value* |
| Age, years |  | 29.3 (2.6) | 32.7 (0.8) | 0.01 |
| Gestational age, completed weeks |  | 39 (0.8) | 36.9 (3.5) | 0.12 |
| BMI, kg/m^2^ |  | 35.3 (8.1) | 28.7 (5.0) | 0.01 |
| Waist circumference, cm |  | 100.5 (20.1) | 88.0 (9.7) | 0.02 |
| Hip circumference, cm |  | 121.8 (18.6) | 108.6 (10.6) | 0.02 |
| Skeletal muscle mass, kg |  | 30.1 (6.9) | 28.2 (3.8) | 0.2 |
| Body fat percentage, % |  | 44.3 (8.2) | 36.1 (7.7) | 0.03 |
| Visceral fat area, cm^2^ |  | 190.2 (62.6) | 127.74 (44.7) | 0.01 |
| Systolic BP, mmHg |  | 124 (10) | 117.3 (8.4) | 0.08 |
| Diastolic BP, mmHg |  | 80 (1) | 78 (9) | 0.32 |
| Heart rate |  | 77 (10) | 71 (13) | 0.21 |
| Total cholesterol, mmol/l |  | 4.1 (1.0) | 4.6 (0.7) | 0.9 |
| LDL cholesterol, mmol/l |  | 2.9 (0.9) | 3.0 (0.7) | 0.65 |
| HDL cholesterol, mmol/l |  | 1.1 (0.3) | 1.4 (0.4) | 0.95 |
| Triglycerides, mmol/l |  | 1.4 (0.6) | 1.4 (1.0) | 0.45 |
| HbA1c mmol/mol |  | 35.8 (2.7) | 34.9 (3.4) | 0.32 |
| Total carotenoids^a^, µmol/l |  | 1.38 (0.2) | 1.79 (0.1) | 0.13 |
| The data are presented as mean and standard deviation. *independent Student’s t-test  BMI=body mass index; BP=blood pressure; HbA1c=glycated hemoglobin; HDL=high-density lipoprotein; LDL=low-density lipoprotein ^a^ Carotenoids (lutein, zeaxanthin, β-cryptoxanthin, α-carotene, β-carotene, and lycopene) were analyzed among 4 dropouts and 39 women who completed the study. One sample was excluded for not following the specified protocol for sample preparation. | | | | |
